# Thalidomide as a rescue protocol for treatment of multiple myeloma in dogs: preliminary data from a multicentre retrospective study

**DOI:** 10.3389/fvets.2025.1695122

**Published:** 2026-01-22

**Authors:** Stefano Ciccarelli, Chiara Leo, Chiara Perrone, Delia Franchini, Irene Bonazzi, Riccardo Finotello

**Affiliations:** 1 Department of Veterinary Medicine, University of Bari Aldo Moro, Bari, Italy; 2 Anicura Istituto Veterinario di Novara, Granozzo con Monticello, Novara, Italy; 3 Anicura Ospedale Veterinario “i Portoni Rossi”, Zola Predosa, Bologna, Italy

**Keywords:** canine, multiple myeloma, plasma cell tumors, repurposed drug, thalidomide

## Abstract

Multiple Myeloma (MM) in dogs is typically treated with a combination of alkylating agents and corticosteroids. However, treatment failure or intolerance, often due to cumulative toxicities, can limit the long-term efficacy of these protocols. Thalidomide, an immunomodulatory and anti-angiogenic drug widely used in human MM, remains largely unexplored in veterinary oncology. This study retrospectively evaluated the clinical efficacy and safety of thalidomide as a rescue therapy in dogs with MM that were refractory to or intolerant of standard treatments. Medical records from three referral centers were reviewed, identifying dogs that met the inclusion criteria. All seven selected dogs received melphalan, and four were also treated with cyclophosphamide prior to thalidomide. Thalidomide was administered once daily in all dogs, with one case requiring dose escalation. The median duration of thalidomide administration was 440 days (range: 146–580 days). A complete response was achieved or maintained in five dogs (71%). Adverse events (AEs) were limited to grade II lethargy in two cases, with no hematologic, gastrointestinal, or urinary AEs reported. The median PFS during thalidomide treatment was 490 days (95% CI: 408.7–571.4), while it was 180 days (95% CI: 54.8–305.2) during melphalan therapy. Median overall survival (OS), calculated from diagnosis to last follow-up, was 680 days (95% CI: 542.8–817.2). These findings suggest that thalidomide is a well-tolerated and potentially effective rescue therapy for canine MM, particularly in patients unresponsive to or unable to tolerate conventional chemotherapy. Further prospective studies are warranted to evaluate its efficacy as part of first-line or combined protocols.

## Introduction

Multiple myeloma (MM) is a haematologic malignancy characterized by the systemic clonal proliferation of plasma cells or their precursors, which produce immunoglobulins (1). In dogs, MM accounts for approximately 8% of haematopoietic tumors and 3.3% of bone tumors (2, 3).

Diagnosis is typically based on the identification of at least two of the following criteria: (1) bone marrow plasmacytosis, (2) osteolytic bone lesions, (3) monoclonal gammopathy (M-component), and (4) Bence-Jones proteinuria (4, 5). Although MM is generally considered incurable, chemotherapy can substantially reduce tumor burden, alleviate clinical signs, and lower serum immunoglobulin concentrations, thereby improving both quality and duration of life in most affected dogs (4). Alkylating agents in combination with glucocorticoids are the standard of care (6). Melphalan (L-phenylalanine mustard), a nitrogen mustard derivative (bimustard chloroethylamine), exerts its antitumour effect primarily through DNA alkylation, ultimately causing DNA damage and cell death (3, 4, 7). It can be administered either as a continuous daily dose or in a pulse-dosing regimen (6, 8). For enhanced efficacy, melphalan is typically given concurrently with glucocorticoids (6).

Treatment with melphalan and glucocorticoids has been associated with a median survival time (MST) of 540 days and an overall response rate (OR) of 92% (3, 4). The principal adverse effect of melphalan is myelosuppression, particularly delayed thrombocytopenia, which may recover slowly and can be irreversible in some cases (4, 9). In the event of significant myelosuppression, melphalan should be discontinued and reintroduced at a reduced dose once bone marrow recovery is evident (4). Alternatively, melphalan may be substituted with another alkylating agent, such as cyclophosphamide (3, 7, 10).

Cyclophosphamide toxicity is primarily characterised by neutropenia (dose-limiting) and gastrointestinal side effects (11). Additionally, sterile haemorrhagic cystitis has been reported in up to 34% of dogs, depending on the dosage schedule and duration of treatment (12).

Despite treatment advances, long-term prognosis remains poor, mainly because of the high relapse rate and the frequent development of drug-resistant disease (13).

Thalidomide (α-N-[phthalimido] glutarimide) and its more recently developed synthetic derivatives such as lenalidomide and pomalidomide, have significantly changed the therapeutic landscape of MM in humans due to their antineoplastic, antiangiogenic and immunomodulatory effects (14–16). In human these agents are now integral components of first-line chemotherapy protocols and have significantly improved outcomes in individuals with this malignancy (17). Several studies have investigated the use of thalidomide in veterinary oncology both as monotherapy and in combination with surgery, traditional chemotherapy and metronomic chemotherapy regimens (18–22). Thalidomide has been administered to dogs with mammary tumours (19), haemangiosarcoma (HSA) (20) and pulmonary carcinoma (21) with data suggesting a possible positive role in delaying metastatic disease, extending survival times and reducing tumour vascularisation. Pharmacokinetic and toxicity studies in healthy dogs have shown that thalidomide is well tolerated ad doses up to 1,000 mg/kg with no genotoxic effects (23). In client owned dogs, daily doses, ranging from 5 to 10 mg/kg, have been associated with acceptable adverse event (AE) profiles, primarily sedation and peripheral neurologic signs which appear dose-dependent and cumulative. Despite these effects, thalidomide displays a wide safety margin, although its maximum tolerated dose in dogs remains to be established (24).

To the best of our knowledge, the use of thalidomide in dogs with MM has been reported anecdotally by some authors (4), but no study has yet been conducted to evaluate its efficacy. The primary aim of this retrospective study was to evaluate the efficacy and safety of thalidomide as an alternative treatment in dogs with MM, following disease progression or the development of unacceptable AEs during first- or second-line therapy.

## Materials and methods

Medical records from three referral centres [Cancer Care Unit, Department of Veterinary Medicine, University of Bari (Valenzano, Bari, Italy); Oncology Unit, AniCura Portoni Rossi Veterinary Hospital (Zola Predosa, Bologna, Italy); and Oncology Unit, Novara Veterinary Institute (Novara, Italy)] were retrospectively reviewed to identify dogs diagnosed with MM between March 2022 and January 2025.

The study was approved by the CER (Research Ethics Commitee, n. protocol 132995) of the University of Bari Aldo Moro.

Inclusion criteria required a confirmed diagnosis of MM, based on the presence of at least two of the following findings: (1) clonal gammopathy, (2) osteolytic bone lesions, (3) bone marrow plasmacytosis, and (4) Bence-Jones proteinuria. Additionally, dogs must have received thalidomide following either disease progression or adverse events graded ≥3 according to the VCOG-CTCAEv2 criteria during first- or second-line therapy (25). Cases were included only if complete medical records and sufficient follow-up were available to enable objective assessment of treatment response and adverse effects. Dogs were excluded if medical documentation was incomplete, if thalidomide was discontinued before any follow-up evaluation, or if no post-treatment data were available to determine clinical outcome.

Data retrieved from clinical records included sex and neuter status, breed, age, weight, clinical examination findings, and laboratory test results [complete blood count (CBC), serum biochemistry, serum protein electrophoresis, urinalysis including proteinuria assessment, coagulation profile, and bone marrow aspiration]. Imaging findings from survey radiographs, abdominal ultrasonography, or total body computed tomography (TBCT) were also recorded.

Further information collected encompassed drug dosage, treatment protocol, duration, response, and AEs associated with first-line treatment (melphalan and glucocorticoids) and, where applicable, second-line therapy (cyclophosphamide). Treatment duration, response, and AEs were similarly assessed for a thalidomide therapy.

The response rate was assessed based on the adapted Veterinary Cooperative Oncology group's RECIST response guideline for solid tumours (5, 26) through physical examination and evaluation of CBC, serum electrophoresis and urinalysis in all patients. Depending on the clinical presentation, ionised calcium levels and imaging were also evaluated. Re-evaluations were intended every 3–4 weeks, with actual timing determined by the clinician's discretion. Responses were defined as follows: a complete response (CR) was characterized by the disappearance of all measurable lesions on imaging, the absence of detectable clonal paraprotein in serum and urine, and the normalisation of any clinical or laboratory abnormalities; partial response (PR) was defined as a reduction of at least 30% in the size of measurable lesions on imaging and a reduction of at least 50% in serum and/or urinary clonal protein levels, along with evident clinical improvement. Stable disease (SD) was considered when changes in the size of the lesion were less than a 30% reduction but did not exceed a 20% increase, and/or when the reduction in clonal protein levels was < 50%, with no appearance of new bone or organ lesions, and overall clinical stability. Progressive disease (PD) was defined by an increase >20% in the size of existing lesions, the development of new lesions or clinical deterioration such as new pathological fractures, severe hypercalcaemia or worsening general condition. Adverse events were classified and graded according to the grading system from the Veterinary Cooperative Oncology Group Common Terminology Criteria for Adverse Events (VCOG-CTCAE v2) (25). Mild AEs were classified as grade < 2, while severe AEs were classified as grade >2.

Due to the small number of cases, statistics were only descriptive. Overall Survival (OS) was calculated from the time of diagnosis to death due to any cause. Progression-Free Survival (PFS) was calculated twice: first from the date of the initial administration of melphalan to the date of disease progression or treatment change; and subsequently from the first administration of thalidomide to the date of documented progression PFS and OS were calculated using the Kaplan–Meier method.

## Results

### Study population

Seven dogs met the inclusion criteria and were enrolled in the study. The mean age was 10.4 ± 1.7 years (range, 8–13 years) with a mean body weight of 20.85 kg (range, 6–35 kg). The cohort consisted of four males (one neutered) and three spayed females.

The most represented breed was mixed breed (*n* = 3), followed by Japanese Akita Inu (*n* = 2), German Shepherd Dog (*n* = 1) and Shi Tzu (*n*=1). Presenting complaints and clinical signs are summarized in Table 1.

**Table 1:** Presenting complaints and clinical findings at diagnosis.

**Observations**	**Number of dogs**	**Percentage of dogs**
Lethargy	5/7	71%
Lameness	4/7	57%
Haemorrhage/bleeding disorders	3/7	43%
Weight loss	3/7	43%
Polyuria/polydipsia	3/7	43%
Anorexia	2/7	29%
Diarrhoea	2/7	29%
Cystitis	1/7	14%
Cutaneous lesions	1/7	14%
Fever	1/7	14%
Ocular abnormalities	1/7	14%

Laboratory abnormalities at the time of the diagnosis are summarised in Table 2.

**Table 2:** Laboratory abnormalities at the time of diagnosis.

**Laboratories findings**	**Number of dogs**	**Percentage of dogs**
***Anaemia (PCV)***		
Mild	4/7	57%
Moderate		
Severe		
Regenerative	0/7	
Non-regenerative	4/7	
***Neutropenia***		
Mild	2/7	29%
Moderate		
Severe	1/7	14%
***Thrombocytopenia***		
Mild	1/7	14%
Moderate	2/7	29%
Severe	1/7	14%
*Hypoalbuminemia*	5/7	71%
ALP (Alkaline Phosphatase), high	4/7	57%
ALT (Alanine aminotransferase), high	2/7	29%
AST (Aspartate aminotransferase), high	2/7	29%
Ionised hypercalcemia	3/7	43%
Hypergammaglobulinemia	6/7	86%
Monoclonal	6/7	86%
Polyclonal	-	-
Proteinuria	3/7	43%
Prolonged PT (prothrombin time) and aPTT (partial thromboplastin time)	2/7	29%
Cytological evidence of cutaneous plasmacytoma	1/7	14%
Bone marrow plasmacytosis	2/7	29%

Severity of laboratory abnormalities refers to the degree of deviation from normal reference ranges at the time of diagnosis. Anaemia: mild = PCV 30–37%; moderate = 20–29%; severe = < 20%. Neutropenia: mild = 1.5–2.9 × 10^9^/L; moderate = 1.0–1.49 × 10^9^/L; severe = < 1.0 × 10^9^/L. Thrombocytopenia: mild = 75–149 × 10^9^/L; moderate = 50–74 × 10^9^/L; severe = < 50 × 10^9^/L.

### Imaging findings

Abdominal ultrasound examination was performed at the time of diagnosis in 6/7, and no relevant abnormalities were observed. Thoracic radiographs were performed at the time of diagnosis in 6/7, including three standard projections. Thoracic radiographic findings included normal thorax in 5/6, osteolytic lesions in 1/6.

Radiographically diagnosed osteolytic lesions were documented in 3/7 (57%) dogs with vertebrae and femoral involvements. In the remaining one dog TBCT was performed and revealed vertebral osteolytic lesion. Imaging findings at the time of diagnosis are summarised in Table 3.

**Table 3:** Imaging findings at the time of diagnosis.

**Imaging findings**	**Number of dogs**	**Percentage of dogs**
**Thoracic radiography** **(*****n*** = **6)**		
Normal	5/6	83%
Osteolytic lesions	1/6	17%
**Localization of osteolytic lesions assessed by complete survey radiographs (*****n*** = **4)**		
Vertebral bodies	1/4	
Spinous process	1/4	
Femur	2/4	
**Total body computed tomography (*****n*** = **1)**		
Vertebral bodies	1/1	
Spinous process	1/1	

Multiple myeloma was diagnosed using the following criteria: one dog had bone marrow plasmacytosis with osteolytic lesions; one had bone marrow plasmacytosis and monoclonal gammopathy; and five had monoclonal gammopathy with osteolytic lesions.

### Treatments

In one dog, a vertebral stabilisation surgery with dorsal laminectomy was performed prior to initiating chemotherapy. All dogs initially received melphalan (7 mg/m^2^ PO once daily for 5 consecutive days every 3 weeks) combined with glucocorticoids. Glucocorticoids were administered exclusively during melphalan treatment and were discontinued thereafter. Four of the seven dogs were subsequently treated with cyclophosphamide (250 mg/m^2^ PO every 2 weeks), two due to adverse events and two due to disease progression. Following PD or AEs during melphalan and/or cyclophosphamide treatment, all dogs received thalidomide at a dose of 7 mg/kg PO once daily. In one case, the thalidomide dose was increased to 14 mg/kg PO once daily in response to disease progression. A galenic preparation of thalidomide was compounded for each patient by the same veterinary pharmacy, ensuring standardized formulation and accurate dosing across all participating institutions to guarantee individualized treatment. To minimise the impact of lethargy induced by thalidomide (19), owners were instructed to administer the drug in the evening. Additionally, given the potential teratogenic effects of thalidomide, owners were advised to wear disposable gloves during drug administration and avoid direct contact with the animal's bodily fluids.

### Treatment response

Clinical and laboratory reassessments were performed every 3–4 weeks, depending on the patient's clinical condition, as previously described. All seven dogs initially received melphalan combined with glucocorticoids as first-line therapy, with a median treatment duration of 150 days (range: 60–450 days). Overall, 3/7 dogs (43%) achieved a complete response (CR), 3/7 (43%) a partial response (PR), and 1/7 (14%) showed progressive disease (PD) while on melphalan. Among the three dogs that achieved CR during melphalan therapy (with total treatment durations of 180, 360, and 450 days), two experienced documented disease relapse prior to transitioning to cyclophosphamide, whereas in the remaining dog melphalan was discontinued due to severe thrombocytopenia despite persistent CR, and the patient was switched directly to thalidomide. Of the three dogs with PR, one continued melphalan in combination with thalidomide to enhance response. In the remaining two, melphalan was discontinued due to drug-related toxicity, and cyclophosphamide was introduced as an alternative alkylating agent.

In the dog with PD while on melphalan, treatment was discontinued due to the combination of hypercalcaemia with an elevated urine protein-to-creatinine ratio, and secondary cystitis. The infection occurred during melphalan and glucocorticoid therapy in the presence of neutropenia, suggesting a treatment-related predisposition to urinary infection. Thalidomide was subsequently initiated.

A total of 4/7 dogs received cyclophosphamide (250 mg/m^2^ PO once weekly) as a second-line treatment, with a median treatment duration of 71 days (range: 14–120 days). Among these, one dog achieved a PR, two experienced stable disease (SD), and one shown PD. In the PR case, cyclophosphamide was discontinued after 120 days due to limited further improvement. In the two SD cases, treatment was stopped after 17 and 60 days because of adverse effects. In the PD case, cyclophosphamide was discontinued after 90 days, and thalidomide was subsequently initiated.

**Figure 1:**
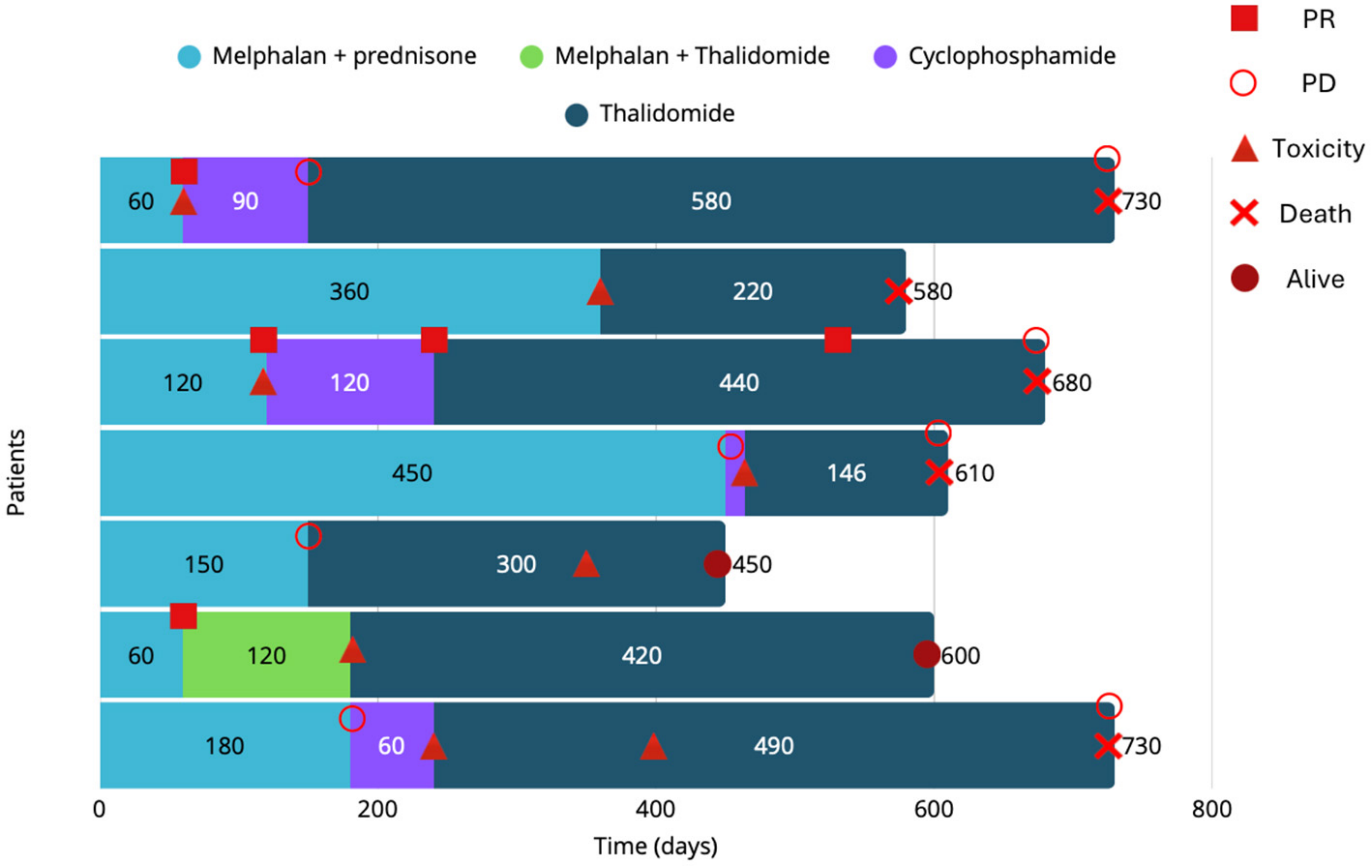
Outcome of treated dogs. Each bar represents 1 dog. The abscissa represents time, and the ordinate represents each case.

Ultimately, all seven dogs received thalidomide in single-agent chemotherapy (initial dose: 7 mg/kg PO SID). At the initiation of thalidomide therapy, two dogs had PD, and three were in PR, of which two also exhibited cyclophosphamide-associated AEs. In the remaining two dogs, thalidomide was introduced due to melphalan-induced thrombocytopenia; one was in CR at the time of the switch, while the other was in PR. Following thalidomide administration, 5/7 dogs (71%) achieved or maintained CR, 1/7 (14%) achieved PR, and 1/7 (14%) showed PD. In the dog initially experiencing a PR, a dose escalation to 14 mg/kg (from the starting dose of 7 mg/kg) was implemented after 3 months due to suboptimal response, resulting in subsequent CR. In the dog with PD, worsening renal function eventually progressed to IRIS stage 4 chronic kidney disease, leading to permanent treatment discontinuation and euthanasia. The treatment course and clinical outcomes for all dogs are showed in Figure 1.

### Other treatments

Supportive analgesic therapy was administered to 4/7 dogs (57%) presenting with lameness. The treatment protocol consisted of paracetamol at 10 mg/kg orally twice daily and gabapentin at 10 mg/kg orally three times daily, continued until clinical signs resolved. Adverse effects such as nausea and vomiting were managed symptomatically with maropitant at 2 mg/kg orally. In dogs presenting with diarrhoea at diagnosis, stool-binding and astringent supplements were administered. In one patient, a frozen whole blood transfusion was required prior to initiating treatment.

### Outcome

At the end of the follow-up period, two dogs were still alive and were therefore censored from the survival analysis. Of the remaining five dogs, four deaths were disease-related according to our response criteria, while one dog died due to chronic kidney disease without documented progression.

For the Overall Survival (OS) analysis, all seven cases were included. Of these, two (28.6%) were still alive at the end of the follow-up period and were therefore censored, while five deaths were recorded. The median OS was estimated at 680 days (95% CI: 542.8–817.2 days). The mean OS was 673.9 ± 27.7 days (95% CI: 619.6–728.2 days).

For PFS during thalidomide treatment, all seven cases were included. Of these, three (42.86%) were censored, and four progression events were recorded. The median PFS for the thalidomide group was 490 days (95% CI: 408.7–571.4 days). The mean PFS was 499.4 ± 63.0 days (95% CI: 408.7–571.4 days).

For PFS during melphalan therapy, six out of the seven cases were included in the analysis, with one dog (16.7%) censored and five progression events recorded. The median PFS for the melphalan group was 180 days (95% CI: 54.8–305.2 days). The mean PFS was 239.2 ± 71.4 days (95% CI: 99.2–379.1 days).

**Figure 2:**
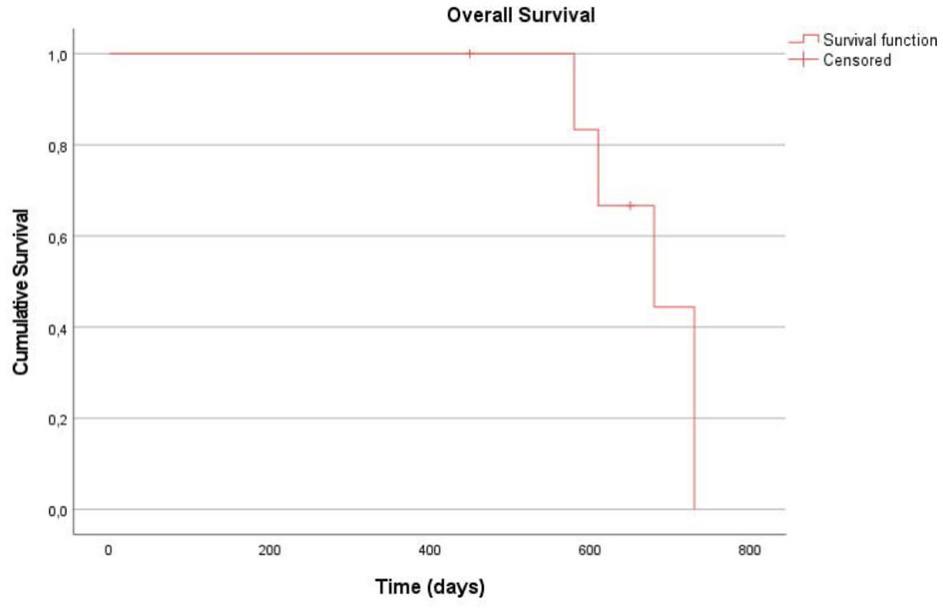
Kaplan-Meier curve for Overall Survival (OS) of the entire cohort (7). The Y-axis represents the cumulative probability of survival, and the X-axis indicates time in days. The tick marks along the curve represents censored observations.

**Figure 3:**
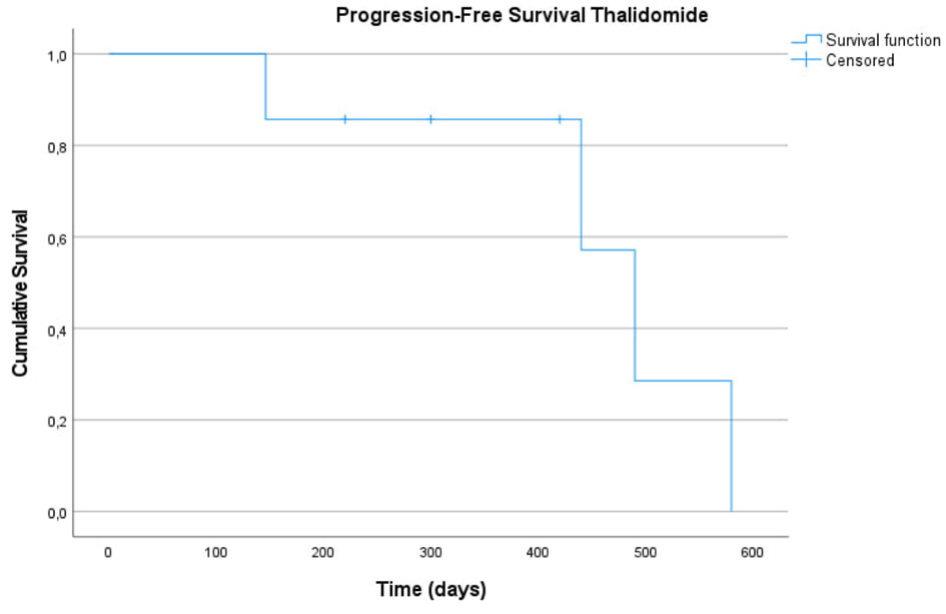
Kaplan-Meier curve for Progression-Free Survival (PFS) under the Thalidomide treatment (7). The Y-axis represents the cumulative probability of remaining free from disease progression, and the X-axis indicates time in days. The tick marks along the curve represents censored observations.

**Figure 4:**
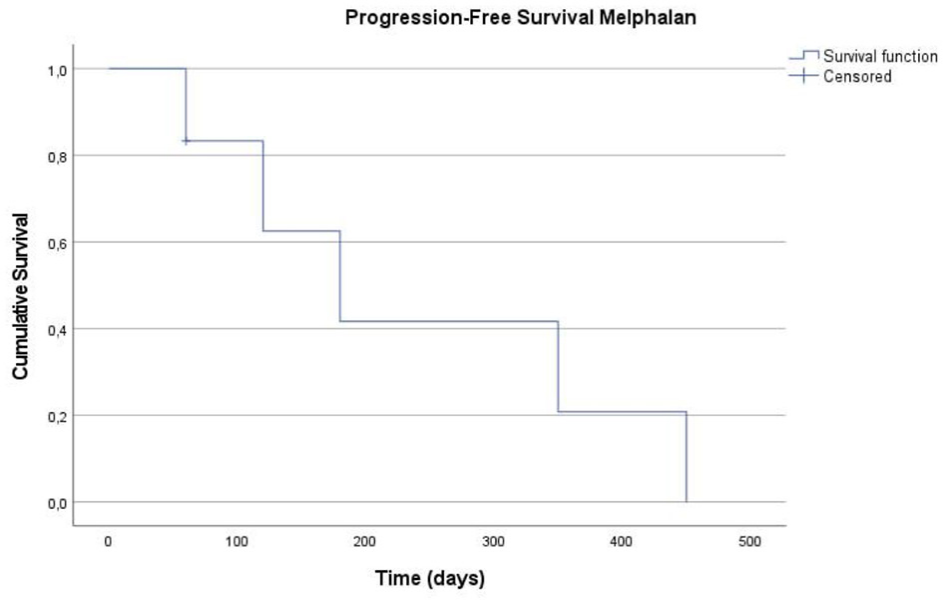
Kaplan-Meier curve for Progression-Free Survival (PFS) under the Melphalan treatment (7). The Y-axis represents the cumulative probability of remaining free from disease progression, and the X-axis indicates time in days. The tick marks along the curve represents censored observations.

Kaplan–Meier curves for OS and PFS during both treatments are presented in Figures 2–4.

### Adverse events

Chemotherapy-related AEs are summarized in Table 4. Hematologic AEs were observed in 4/7 dogs (57%) treated with melphalan, including grade IV thrombocytopenia in 2/7 dogs. Cyclophosphamide administration was associated with gastrointestinal and urinary AEs in 4/7 dogs (57%) including grade III-IV vomiting in 3/7 and grade III cystitis in 1/7. Thalidomide-related AEs were limited to lethargy which occurred in 2/7 dogs (29%) as a grade II event.

**Table 4:** Adverse Events associated with chemotherapy administration in dog population according to VCOG (v2) (25).

**Melphalan**	**Number of dogs**	**Percentage of dogs**
Blood/bone marrow toxicity		
**Anaemia (PCV)**		
Grade I		
Grade II		
Grade III	1/7	14%
Grade IV	1/7	14%
**Neutropenia**		
Grade I		
Grade II		
Grade III	1/7	14%
Grade IV		
**Thrombocytopenia**		
Grade I		
Grade II		
Grade III	1/7	14%
Grade IV	2/7	28%
Cyclophosphamide	Number of dogs	Percentage of dogs
**Gastrointestinal** **Anorexia**		
Grade I	3/7	43%
Grade II		
Grade III	1/7	14%
Grade IV		
**Vomiting**		
Grade I		
Grade II		
Grade III	1/7	14%
Grade IV	2/7	28%
**Cystitis**		
Grade I		
Grade II		
Grade III	1/7	14%
Grade IV		
Thalidomide	Number of dogs	Percentage of dogs
**Constitutional clinical signs**		
**Lethargy**		
Grade I		
Grade II	2/7	28%
Grade III		
Grade IV		

## Discussion

To the best of our knowledge, this is the first study investigating the use of thalidomide as a rescue therapy for canine MM. The cohort consisted of dogs exhibiting resistance or intolerance to standard first- and second-line chemotherapeutic protocols, thus representing a particularly challenging clinical population.

In human medicine, several studies have extensively characterised the bone marrow microenvironment as a critical regulator of multiple myeloma growth, survival, and progression. Malignant plasma cells interact with surrounding stromal and immune cells, inducing the secretion of various pro-tumoral cytokines and growth factors, such as Interleukin-6 (IL-6), VEGF, insulin-like growth factor-1 (IGF-1), transforming growth factor-beta (TGF-β), and stromal cell-derived factor-1 (SDF-1)(27). These factors collectively enhance tumour proliferation, angiogenesis, drug resistance, and evasion of apoptosis (28). Although these mechanisms have not yet been investigated in canine MM, analogous processes could reasonably be hypothesised.

Thalidomide was introduced in human medicine in the late 1990s as a rescue therapy for relapsed or refractory MM (29, 30). Its clinical efficacy led to an initial FDA approval, followed by worldwide acceptance, and inspired the development of more potent immunomodulatory derivatives (IMiDs), such as lenalidomide and pomalidomide (31). These agents exert multiple anti-myeloma effects, including inhibition of nuclear factor kappa-light-chain-enhancer of activated B cells and Interferon Regulatory Factor 4 pathways, promotion of apoptosis (e.g., Caspase-8 upregulation), suppression of angiogenesis via VEGF and IL-6 downregulation, and modulation of tumor–stroma interactions (30, 31). Additionally, IMiDs enhance the immune response by restoring T-cell function, promoting a Th1-mediated antitumour environment, activating dendritic cells, and re-establishing effective immunosurveillance within the immunosuppressive bone marrow microenvironment characteristic of MM (31). Such multifactorial mechanisms account for the wide and synergistic antitumor activity of these agents in human MM, combining antiproliferative, antiangiogenic, and immune-modulating effects that collectively enhance tumour control and delay disease progression.

The main limitation of all veterinary reports on thalidomide, including the present study, lies in the absence of a clearly established mechanism of action in the dog. While the molecular actions of thalidomide and related compounds remained unclear until relatively recently, cereblon (CRBN) was identified as a key direct binding target (32). CRBN functions as a ligand-dependent substrate receptor within the cullin-RING E3 ubiquitin ligase complex (CRL4CRBN). Upon binding of thalidomide or similar ligands, CRBN can recruit different “neosubstrates” such as IKF1 and Aiolos, with substrate recognition largely dictated by the structural features of the ligand (32). However, amino acid differences within canine CRBN closely resemble those described in mice, where they abolish this canonical CRBN-dependent pathway, thereby questioning its relevance in dog. Nevertheless, thalidomide exerts additional CRBN-independent effects, including anti-angiogenic and immunomodulatory activity via modulation of VEGF, FGF2 and TNF-α (33). In support of this, reduced VEGF expression has been observed in hemangiosarcoma metastases from dogs treated with thalidomide (20). Furthermore, the zinc finger 2 domain of the Sal-like protein 4 (SALL4), a potential neosubstrate implicated in thalidomide's teratogenicity, is more conserved between dogs and humans than between humans and mice, raising the possibility that a CRBN–SALL4–mediated effect could still occur in some canine cells (34). Taken together, these biological observations provide a rationale for the clinical activity seen in this cohort, where thalidomide may exert antitumor effects in canine multiple myeloma through one or more of these non-classical pathways. Importantly, the absence of typical toxicities in dogs should not be regarded as evidence of inefficacy, but rather as a species-specific pharmacodynamic feature. While further mechanistic and prospective studies are warranted, these findings support the cautious but continued exploration of thalidomide as a therapeutic option in canine oncology.

Several studies have already explored the clinical use of thalidomide in veterinary oncology. In dogs with advanced-stage mammary gland carcinomas, thalidomide was generally well tolerated, with AEs limited to mild and reversible sedation (19). In a retrospective study evaluating metronomic chemotherapy for advanced pulmonary carcinoma in dogs, thalidomide (administered alongside cyclophosphamide and piroxicam) was associated with improved time to progression and overall survival and was linked to improved quality of life (21). Similarly, in canine HSA, the addition of thalidomide to metronomic chemotherapy, following standard adjuvant protocols, was associated with prolonged time to metastasis and survival, suggesting a potential synergistic effect between traditional and metronomic regimens (35).

These findings provide preliminary evidence that thalidomide's anti-angiogenic properties may also be active in canine MM, potentially contributing to delayed tumour progression. We hypothesised that thalidomide might therefore provide clinical benefit in dogs unable to tolerate or resistant to melphalan or cyclophosphamide.

Melphalan combined with glucocorticoids remains the standard treatment for canine MM. In our cohort, three dogs achieved a complete response and three a partial response. This outcome likely reflects the refractory nature of the population studied rather than a failure of first-line therapy itself.

Notably, two dogs developed severe thrombocytopenia after prolonged melphalan administration, requiring drug discontinuation. Both were Japanese Akita Inu, a breed predisposed to hereditary macrothrombocytopenia, which may appear as a physiological finding despite reduced platelet counts, emphasising the need for breed-specific treatment considerations (36, 37).

Cyclophosphamide was used as a second-line treatment in four dogs due to melphalan-associated disease progression or AEs. Although one dog achieved a PR and two exhibited SD, cyclophosphamide was ultimately discontinued in all cases, either due to limited clinical benefit or the development of severe toxicities, including grade III anorexia, grade IV vomiting, and haemorrhagic cystitis. Although severe gastrointestinal adverse events are generally uncommon with cyclophosphamide, their high incidence in our population likely reflects the inclusion of dogs that were already refractory to or intolerant of standard chemotherapy. Consequently, these cases may have shown increased susceptibility to treatment-related toxicity rather than experiencing an unexpected drug-specific effect.

Thalidomide was introduced as a rescue treatment in all seven dogs following either suboptimal response or treatment-limiting toxicity to previous therapies. In six dogs, it was initiated after partial response, stable disease, or AEs associated with melphalan or cyclophosphamide. In one dog, thalidomide was introduced immediately after melphalan discontinuation, as disease progression combined with bacterial cystitis limited the feasibility of cyclophosphamide as a second-line treatment. Despite treatment, this patient experienced progressive renal decline and was subsequently euthanised.

Despite the refractory nature of the study population, thalidomide demonstrated significant clinical efficacy. A CR was achieved or maintained in 5/7 dogs (71%), while in one dog an initial partial response (PR) improved to CR following a dose escalation from 7 to 14 mg/kg/day. Only one patient failed to respond; notably, this dog had already developed renal failure, which continued to progress despite thalidomide administration.

Thalidomide was well tolerated in our cohort, with only two dogs experiencing grade II lethargy. However, since owners were instructed to administer the drug in the evening to minimize its sedative effects, this adverse event may have been underreported. No haematologic, gastrointestinal, or urinary AEs were observed throughout the treatment period. In one case, thalidomide administration was continued even after achieving CR, reflecting both the absence of significant AEs and the intent to maintain long-term disease control following prior relapse.

These findings are consistent with previous veterinary studies reporting a low incidence of thalidomide-associated AEs in dogs (21, 23, 24, 35, 38). In contrast, thalidomide use in human patients is frequently limited by severe toxicities, including peripheral neuropathy, gastrointestinal disorders, thromboembolism, and dermatologic reactions (39).

The divergent safety profiles observed between species may reflect differences in drug metabolism, receptor distribution, or owner-perceived tolerability in companion animals (23, 38). The favourable AEs profile observed in our study supports the use of thalidomide as a safe and promising therapeutic option, particularly in dogs with poor tolerance to alkylating agents or comorbidities that limit the feasibility of conventional, dose-intensive chemotherapy protocols.

Based on prior literature, MST for dogs treated with melphalan and glucocorticoids range from 540 days [4, 41] to as high as 930 days in more recent studies (6). Given the refractory or intolerant status of our patients, a substantially shorter survival time could have been expected. Notably, in our cohort, median overall survival (OS), calculated from diagnosis to last follow-up and therefore encompassing the entire treatment course, was 680 days (95% CI: 542.8–817.2). Median progression-free survival (PFS) during thalidomide treatment was 490 days (95% CI: 408.7–571.4 days), while PFS during melphalan treatment was 180 days (95% CI: 54.8–305.2). These findings suggest that thalidomide may significantly contribute to prolonging survival even in a pretreated population, supporting its potential role as a viable rescue therapy in canine MM.

The small sample size represents a major limitation of this study, as it reduces the statistical power and limits the generalisability of the findings. In addition, treatment protocols were not standardised across cases, reflecting the retrospective and multi-institutional design. As a result, the calculated OST should be interpreted with caution, since it may not fully reflect the specific contribution of individual drugs or treatment sequences. Another limitation concerns the absence of standardised haematologic monitoring intervals and the short timeframes between treatments, which may have obscured subtle or cumulative hematologic effects attributable specifically to thalidomide. Further prospective investigations adopting uniform monitoring schedules and defined washout periods are warranted to clarify these aspects. The use of VCOG-RECIST criteria (26), originally developed for the assessment of solid tumours, also represents a methodological limitation when applied to a haematologic malignancy such as multiple myeloma. Nevertheless, these criteria were adapted in the present study to allow for the most consistent and objective clinical evaluation possible within a retrospective framework, ensuring comparability across cases. Moreover, this study did not explore potential correlations between treatment response and patient-related factors such as age, sex, breed, or clinical presentation. Pharmacogenetic variations, including breed-specific mutations, could influence therapeutic efficacy and toxicity, highlighting a potential avenue for future personalised approaches. Finally, despite the promising clinical outcomes observed, this study should be regarded as preliminary. Prospective studies with larger cohorts, standardised treatment protocols, and extended follow-up are required to better define the clinical utility, safety profile, and therapeutic role of thalidomide—both as a rescue therapy and as a potential component of first-line or combination regimens for canine multiple myeloma.

## Conclusions

This is the first study to investigate the use of thalidomide as a rescue therapy for dogs with MM who are either refractory to or intolerant of standard chemotherapy protocols. Despite the advanced and refractory nature of the cohort, thalidomide demonstrated notable clinical efficacy, achieving a complete response rate of 71% with minimal toxicity. Given its anti-angiogenic and immunomodulatory properties, thalidomide appears to be a promising alternative when conventional alkylating agents are ineffective or contraindicated due to AEs. These results provide a rationale for further investigating thalidomide not only as a rescue option, but also as a possible component of first-line or combination protocols in the management of canine MM.

Prospective studies with larger sample sizes, standardised monitoring and exploration of pharmacogenetic influences are needed to better define the clinical utility and therapeutic application of thalidomide in treatment strategies for canine MM, including its potential role in earlier treatment setting or in combination protocols.

## Data Availability

The original contributions presented in the study are included in the article/supplementary material, further inquiries can be directed to the corresponding author.
